# Sexual Satisfaction Mediates the Effects of the Quality of Dyadic Sexual Communication on the Degree of Perceived Sexual Desire Discrepancy

**DOI:** 10.3390/healthcare11050648

**Published:** 2023-02-23

**Authors:** Roberta Galizia, Annalisa Theodorou, Chiara Simonelli, Carlo Lai, Filippo Maria Nimbi

**Affiliations:** 1 Department of Dynamic, Clinical and Health Psychology, Sapienza University of Rome, 00185 Rome, Italy; 2 Department of Education, Roma Tre University, 00185 Rome, Italy

**Keywords:** perceived sexual desire discrepancy, romantic relationships, sexual communication, sexual satisfaction

## Abstract

Difficulties related to sexual desire discrepancy are among the most common complaints in people seeking help in therapy. The aims of the current study were to test a mediation model using a bootstrapping procedure where the quality of the dyadic sexual communication plays a key role in increasing or diminishing the degree of perceived sexual desire discrepancy through sexual satisfaction. An online survey was administered through social media to N = 369 participants involved in a romantic relationship, measuring the quality of dyadic sexual communication, sexual satisfaction, the degree of the perceived sexual desire discrepancy, and a series of relevant covariates. As expected, the mediation model showed that a better quality of dyadic sexual communication is related to a lower degree of perceived sexual desire discrepancy through increased sexual satisfaction (β = −0.17, SE = 0.05, [95% CI = −0.27, −0.07]). The effect held over and above the effect of the relevant covariates. The theoretical and practical implications of the present study are discussed.

## 1. Introduction

Researchers, clinicians, and educators are interested in sexual desire because it affects both intimate relationships [1,2] and the individual [3]. Difficulties related to sexual desire discrepancy are one of the most common complaints among people seeking help in therapy [4,5,6,7]. Sexual desire discrepancy was first defined by Zilbergeld and Ellison [8] to describe when two partners engaged in an intimate relationship desire different levels or different frequencies of sexual activity. However, the frequency of sexual activity is not a good indicator of desire, as people have sex for different reasons (e.g., pleasing a partner, relieving stress, routine) and often without experiencing sexual desire [5,9,10]. In addition, sex may mean different things to different people [5,9,11], suggesting that sexual desire discrepancy might represent a lack of motivation to engage in specific sexual activities with a specific partner [5,12]. To date, many studies [1,2,5,13] have referred to sexual desire discrepancy as the difference between both partners’ scores on sexual desire (i.e., *actual* sexual desire discrepancy); others, instead, have explored the “*perceived* sexual desire discrepancy” by asking each partner to provide an estimate of how discrepant their sexual desire is [5,14]. However, scientific research on sexual desire discrepancy is still scarce and complicated, as it is characterized by conceptual and methodological problems that make it difficult to define, measure, compare results, and treat it [5].

In order to develop effective clinical guidelines and treatment protocols to support people who complain of distress caused by sexual desire discrepancy, sexual health researchers and clinicians need to delve deeper into the determinants and processes underlying it. Finally, studies have mainly focused on associations between sexual desire discrepancy and sexual and relational satisfaction [15,16,17], leaving out other important relational aspects, such as sexual communication [5]. Sexual communication was defined by Metts and Cupach [18] as the integration of sexual self-report, the quality of sexual communication, and the frequency of communication. Sexual self-report includes the communication of sexual preferences and the desire to engage in specific sexual activities [19], as well as sexual values, past experiences, and sexual attitudes [20]. The frequency of sexual communication indicates how often couples discuss different aspects of their sexual relationship. The quality of sexual communication has been conceptualized as the integration of satisfaction with sexual communication [21], the perception of being able to talk with a partner about the positive and negative aspects of the sexual relationship [22], and the emotional safety of communicating about sexual matters [23]. Research has already shown that sexual communication is an important protective factor for the development and maintenance of sexual health outcomes, such as sexual satisfaction and desire [24,25,26,27]. Indeed, studies have highlighted that couples who experience sexual difficulties and show sexual dissatisfaction may hide problems with sexual communication [27,28], and that communication between partners about sexual desire discrepancy may prove to be a way to increase desire levels [16,29]. 

### 1.1. The Beneficial Role of Sexual Communication in Sexual Satisfaction

Scientific research has now amply demonstrated that communication within relationships is associated with greater sexual satisfaction [30,31,32], which is defined as “an affective response resulting from the subjective evaluation of positive and negative dimensions associated with one’s sexual relationship” [33] (p. 268). In particular, communicating more frequently about sexual issues with one’s partner and perceiving the better quality of dyadic communication are associated with both higher levels of sexual satisfaction and greater relationship satisfaction [34]. Also in the model proposed by Cupach and Comstock [31], satisfaction with sexual communication appears to lead to sexual satisfaction, and the latter seems to contribute to dyadic adjustment in marital relationships. Moreover, in line with some studies [35,36,37], open communication between partners on sexual matters (e.g., sharing one’s sexual preferences) would increase closeness between couples as well as intimacy, and the likelihood that partners would fulfill their sexual desires.

### 1.2. The Effect of Sexual Communication and Sexual Satisfaction on Sexual Desire 

As reported in the previous section, numerous studies have explained how sexual communication is associated with sexual satisfaction [30,31,32], which, in turn, is considered an important aspect of sexual function [27]. Communicating one’s sexual preferences to one’s partner can lead to greater sexual gratification, as well as pursuing one’s sexual desires and pleasures and avoiding what one does not like. Greater sexual communication may be important precisely because sexual preferences may differ between partners and change over the course of one’s life experience [27,38]. Sexual communication can, therefore, be helpful in modifying and enhancing sexual experience and intimacy, positively influencing sexual satisfaction and sexual function. In contrast, reduced or absent dyadic communication about one’s sexual preferences can lead to sexual difficulties [27]. In fact, couples who report worse communication quality often have sexual problems [37,39,40]. Indeed, numerous studies have reported that in the presence of better dyadic communication, couples feel sexually satisfied for longer in the face of changing sexual preferences and sexual difficulties [23,35,36,41,42,43]. It follows that more and better sexual communication leads to greater emotional intimacy, which in turn facilitates greater sexual satisfaction and better sexual function [27,44]. Several studies have found a strong link between sexual satisfaction and sexual desire, showing that couples who are more sexually satisfied also perceive greater sexual desire than those who are less sexually satisfied [1,2,45]. This evidence may be explained by the fact that sexual desire is an emotional–motivational response triggered by rewarding signals that individuals perceive as pleasurable and satisfying [5]. Thus, feeling more satisfied with sex may increase the desire to have sex. A recent systematic review conducted by Mark and Lasslo in 2018 [45] highlighted that sexual satisfaction is an important protective predictor of sexual desire in long-term relationships, and that sexual dissatisfaction and dissatisfaction related to the quality of communication with one’s partner are risk factors for the maintenance of sexual desire. In fact, differences in sexual desire between partners occur more in long-term relationships [5,15,45] and as a way to express sexual and/or relational dissatisfaction [1,2,5,17]. Research has also shown a stronger link between sexual satisfaction and sexual desire than between relational satisfaction and sexual desire [1,2], probably because of the sexual nature shared by both [45]. Moreover, a meta-analysis [27] conducted on 48 studies published between 1980 and 2017 showed that sexual communication is positively associated with all domains of sexual function, including sexual desire. The results revealed that sexual communication plays a key role in facilitating sexual desire, especially in women. The results were also supported by a previous study conducted by Ferreira et al. [44], who found that communicating with one’s partner about one’s relationship increases closeness between partners, as well as sexual desire. Interestingly, in a study by Murray et al. [26], most men reported that intimate communication was necessary to achieve sexual desire. Based on this assumption, it is possible to hypothesize that good communication between members of a couple is an essential factor for sexual satisfaction and, in turn, also for sexual desire. 

### 1.3. Aims and Hypothesis

The aims of the current study were to test a mediation model where both the quality of the dyadic sexual communication and sexual satisfaction play a key role in increasing or diminishing the degree of perceived sexual desire discrepancy in individuals involved in a romantic relationship. 

First, based on the literature on sexual desire [27], we expect that a better quality of dyadic sexual communication positively predicts a lower degree of perceived sexual desire discrepancy. Moreover, as previous research has also reported [27,44], communication about sexual issues is an important factor affecting sexual satisfaction. Thus, second, we expect that a better quality of dyadic sexual communication positively predicts sexual satisfaction. As mentioned earlier, people who perceive a better quality of dyadic sexual communication are expected to develop higher levels of sexual satisfaction and, consequently, better sexual response functioning than those who perceive a worse quality of dyadic sexual communication with their partner [1,2,5,45]. Third, we pose that sexual satisfaction positively predicts a lower degree of perceived sexual desire discrepancy. If these assumptions are correct, then it follows that people perceiving a better quality of dyadic sexual communication would perceive a lower degree of perceived sexual desire discrepancy because of their higher levels of sexual satisfaction. We expect these effects to be significant beyond the effect of the following selected and most widely used covariates in sex research and, more specifically, sexual desire and sexual desire discrepancy [5,15,45,46,47]: gender, age, sexual orientation, relationship duration, having a child/children, desire to have a child/children, and social desirability bias.

## 2. Materials and Methods

### 2.1. Participants and Procedure

A convenience group participated in the study by filling out an online survey through a snowball technique on social media platforms such as Facebook, Instagram, and WhatsApp. Individuals could complete the online survey by smartphone, personal computer, or tablet. Participants were assured anonymity and, to gain access to the survey, they needed to provide their informed consent to voluntarily take part in the research. The inclusion criteria were being at least 18 years old, being in a stable relationship for at least 1 year, not having had children in the past year and not expecting to have children at the moment, and speaking fluent Italian. 

The study was part of a wider research project aimed at exploring the psycho-relational factors of sexual desire. The data were collected from December 2021 to October 2022. The study was approved by the Ethics Committee of the Department of Dynamic, Clinical and Health Psychology of Sapienza University of Rome (protocol code: 0001543; date of approval: 11 October 2021), and was held according to the Helsinki declaration standards.

There were 369 participants, of which 293 (79.4%) were cisgender women and 76 (20.6%) were cisgender men. Age ranged from 18 to 66 (M = 28.96, SD = 8.63). Of the total sample, 299 (81%) were heterosexual and 70 (19%) were not heterosexual. A total of 220 (59.6%) were not in a cohabiting relationship, 146 (39.6%) were in a cohabiting relationship, and 3 (0.8%%) were in a polyamorous relationship. The duration in years of the relationship ranged from 1 to 40 (M = 6.03, SD = 6.29). Most of the sample (312; 84.6%) had no children, whereas 57 (15.4%) had at least one child. However, 263 (71.3%) desired to have a baby, whereas 106 (28.7%) did not have this desire. Regarding educational qualification, 172 (46.6%) held a degree or equivalent qualification, whereas 144 (39%) held a high school diploma, 49 (13.3%) held a higher degree qualification, and 4 (1.1%) held a junior high school diploma. Regarding employment status, 190 (51.5%) were workers, 134 (36.3%) were students, 42 (11.4%) were unemployed, and 3 (0.8%) were retired.

### 2.2. Measures

#### 2.2.1. Quality of Dyadic Sexual Communication

The Dyadic Sexual Communication Scale (DSC) [48] is a 13-item scale measuring how respondents perceive the discussion of sexual issues with their partners. Items are rated on a 6-point Likert-type scale ranging from 1 (disagree strongly) to 6 (agree strongly). Scores are summed up for a total score that ranges from 13 to 78. A higher score is indicative of a better quality of dyadic sexual communication. The original DSC has shown adequate psychometric properties, including good internal consistency (Cronbach’s alpha = 0.81 total sample, 0.83 cohabiting couples), adequate test–retest reliability (Cronbach’s alpha = 0.89), as well as good discriminant validity between people with and without sexual problems (*p* = 0.001) [48]. The translation into Italian was carried out by the authors, according to the formal rules of the method of forwards and backwards translation. In the present sample, Cronbach’s alpha was 0.89.

#### 2.2.2. Sexual Satisfaction

The Sexual Satisfaction Scale (SSS) [23] is a 30-item measure of sexual satisfaction composed of 5 factors: contentment, communication, compatibility, relational, and personal concern. It showed good psychometric properties and discriminative capability between clinical and nonclinical subjects. Developed for women, it was translated into Italian and was also used for men [47]. For this study, only the contentment subscale was used, which is accessible by coupled individuals. In this study, Cronbach’s α value for this measure was 0.88.

#### 2.2.3. Degree of Perceived Sexual Desire Discrepancy

In line with the literature [5,49], two items measuring the degree of perceived sexual desire discrepancy were created ad hoc: “How would you rate your overall level of desire?” (i.e., the personal actual sexual desire) and “Using the same scale, how would you rate your partner’s overall level of sexual desire?” (i.e., the personal perceived partner’s sexual desire). The participants were asked to indicate the level of sexual desire with each statement using a scale ranging from 1 (no sexual desire) to 10 (highest level of sexual desire). To examine perceived sexual desire discrepancy, a new variable was constructed to represent the absolute difference between one’s own sexual desire and one’s perception of their partner’s sexual desire. The responses for the new variable ranged from 0 to 10, with 0 indicating no discrepancy, and higher scores reflecting an increasing discrepancy. 

#### 2.2.4. Other Measures

A sociodemographic questionnaire was created ad hoc to collect general information such as age, ethnicity, sex assigned at birth, gender identity, sexual orientation, relationship and marital status, relationship duration, education level, and children. 

The covariate related to social desirability bias was assessed using the Marlowe–Crowne Social Desirability Scale Short Form (MCSDS) [50,51]. The MCSDS is comprised of 13 items that assess socially desirable response tendencies (e.g., “I sometimes feel resentful when I don’t get my way”; “I am sometimes irritated by people who ask favors of me”). The participants rated each item as either True or False. Items are summed to create a global index of social desirability. Cronbach’s alpha in the current study was 0.53. The relatively low Cronbach’s alpha seems to agree with other studies that have used the Italian short version of the MCSDS [51,52,53,54].

### 2.3. Strategic Analysis

To investigate the relationships among the quality of dyadic sexual communication, sexual satisfaction, and the degree of perceived sexual desire discrepancy, we first computed the zero-order correlations among these and sociodemographic variables. Then, to test the role of sexual satisfaction as a mediator of the relationship between the quality of the dyadic sexual communication and the degree of perceived sexual desire discrepancy, a mediation model using the Model 4 of the SPSS Macro Process [55] was tested. According to Baron and Kenny [56], the results from three models were obtained. In the first model, the total effect of the quality of dyadic sexual communication on the degree of perceived sexual desire discrepancy was obtained (path *c*). In the second model, the path *a* of mediation was obtained, namely the effect of the quality of dyadic sexual communication on the levels of sexual satisfaction. In the third and last model, the path *b* was obtained, namely the effect of sexual satisfaction on the degree of perceived sexual desire discrepancy when controlling for the quality of the dyadic sexual communication, and the path *c*, namely the direct effect of the quality of the dyadic sexual communication on the degree of perceived sexual desire discrepancy. Finally, we tested the significance of the indirect effect. We included socio-demographic variables (i.e., gender, age, sexual orientation, relationship duration, having a child/children, desire to have a child/children, and social desirability) in the mediation analysis to control for potential confounding variables in the results of the study. A bootstrapping procedure was used (with 5000 bootstrap samples) to estimate 95% confidence intervals (95% CI). According to Preacher and Hayes [57], a 95% CI that does not include zero provides evidence of a significant indirect effect. It has been suggested that the bootstrapping procedure is the most reliable test for evaluating the effects of mediation models [58]. The 0.05 level of significance was adopted throughout all analyses. 

## 3. Results

### 3.1. Descriptives and Correlations

Descriptive statistics and correlations among variables are reported in Table 1. As expected, the quality of dyadic sexual communication and levels of sexual satisfaction were negatively and significantly associated with the degree of perceived sexual desire discrepancy, and the quality of dyadic sexual communication was positively associated with sexual satisfaction. The quality of dyadic sexual communication was negatively associated with age, relationship duration, and having a child/children, and positively associated with the desire to have a child/children and social desirability. The same associations were found in sexual satisfaction, except for the desire to have a child/children, whose association did not emerge as statistically significant. The degree of perceived sexual desire discrepancy was positively and significantly associated with age and relationship duration, and negatively and significantly related to the desire to have a child/children and social desirability. All in all, the correlations among the main variables, namely dyadic sexual communication, sexual satisfaction, and perceived sexual desire discrepancy, were significant and in the expected direction.

### 3.2. Mediation Model

**Figure 1:**
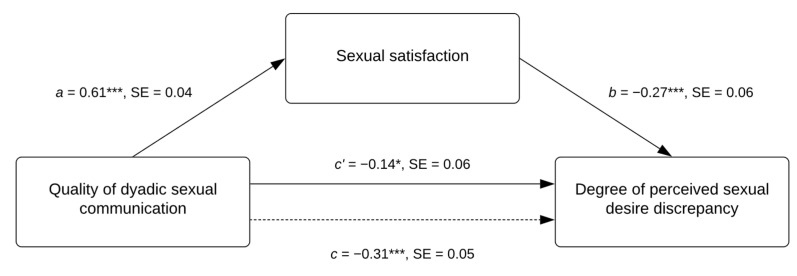
Graphical representation of the results from the mediation model. Please note that the reported estimates were obtained by controlling for the covariates of gender, age, sexual orientation, relationship duration, having a child/children, desire to have a child/children, and social desirability bias. The estimates correspond to standardized effects. The dashed arrow indicates the total effect. * *p* < 0.05; *** *p* < 0.001.

The results of the three models are reported in Table 2. In particular, the total effect was negative and significant, indicating that the quality of dyadic sexual communication was negatively associated with the degree of perceived sexual desire discrepancy (path *c*; see Table 2, Model 1, first column). Moreover, the effect of the quality of dyadic sexual communication on the levels of sexual satisfaction was positive and significant (path *a*; see Table 2, Model 2, third column). Thus, a better quality of dyadic sexual communication was associated with higher levels of sexual satisfaction. In turn, sexual satisfaction was negatively related to the degree of perceived sexual desire discrepancy (path *b*; see Table 2, Model 3, second column). When sexual satisfaction was taken into account, the direct effect of the quality of dyadic sexual communication on the degree of perceived sexual desire discrepancy was weaker, although still significant (path *c’*; see Table 2, Model 3, second column). Finally, the completely standardized indirect effect was negative and significant β = −0.17, SE = 0.05, [95% CI = −0.27, −0.07], ultimately confirming our mediation hypothesis. Regarding the covariates, the effect of the relationship duration was positive and significant on sexual satisfaction (Model 2) and the degree of perceived sexual desire discrepancy (Model 3). Moreover, the effect of social desirability was positive and significant on sexual satisfaction (Model 2). None of the effects of the other covariates were found to be significant. See Figure 1 for a graphical representation of the results of the mediation model.

## 4. Discussion

The present study sought to test whether the quality of dyadic sexual communication plays a key role in increasing or decreasing the degree of perceived sexual desire discrepancy through sexual satisfaction. 

As expected, the results confirmed that a better quality of dyadic sexual communication was associated with a lower degree of perceived sexual desire discrepancy. Furthermore, this effect was mediated by sexual satisfaction. Although the effect of sexual communication on sexual satisfaction and sexual desire is acknowledged by many studies [26,27,32,34,44], the novel findings of the present study concern the testing of this effect on the degree of perceived sexual desire discrepancy. Importantly, this effect held beyond the effect of important covariates such as gender, age, sexual orientation, relationship duration, having children, desire to have children, and social desirability bias.

Regarding the covariates, the effect of relationship duration was positive and significant on sexual satisfaction (Model 2) and the degree of perceived sexual desire discrepancy (Model 3), a result which is supported in previous studies [5,29,45,59]. However, in the correlation, the effect of relationship duration on satisfaction proved to be negative. This artifact may have depended on the co-presence of all variables in the model. In contrast, the effect of relationship duration on the degree of perceived sexual desire discrepancy remained stable and in line with the scientific literature [5,29,45,59]. In addition, the effect of social desirability bias was positive and significant on sexual satisfaction (Model 2), indicating that individuals with higher sexual satisfaction reported a greater tendency to provide a positive self-image. This result is in line with the literature that has reported that those influenced by social desirability bias tend to over-report culturally desired sexual behaviors and under-report sexually undesirable behaviors [60,61,62,63,64], and those studies that emphasize the importance of considering social desirability bias in sexuality research [46,47,48,49,50,51,52,53,54,55,56,57,58,59,60,61,62,63,64,65,66]. However, as reported in Section 2.2.4, this result should be considered with caution given the relatively low Cronbach’s alpha of the measure in this study (i.e., 0.53). Nevertheless, the relatively low Cronbach’s alpha seems to have been found in other studies that used the Italian short version of the MCSDS [51,52,53,54]. None of the effects of the other covariates were significant.

This study has some limitations that should be considered. First, this is a cross-sectional study design; thus, it is not possible to ascertain causality and the direction of the relationships observed. Second, the sample of the study was mainly composed of cisgender women (79.4%); thus, the findings cannot also be representative for cisgender men. Moreover, most studies on sexual desire discrepancy have been conducted on heterosexual individuals and couples, neglecting other sexual orientations [5]. The present study attempted to transcend this limitation by including individuals of non-heterosexual orientation, which included people who identified as bisexual (*n* = 28), homosexual (*n* = 27), pansexual (*n* = 9), asexual (*n* = 2), demisexual (*n* = 3), and sapiosexual (*n* = 1). However, future studies should also include people who identify with different gender identities. Unfortunately, the sample analyzed in the present study consisted only of cisgender individuals (i.e., people who perceive that they belong to the gender in line with their biological sex). In addition, sexual desire discrepancy should be considered a clinically relevant problem when one or both partners are distressed by it, and distress may depend on the importance and meanings partners place on sexual desire [5,29,67,68,69]. Sexual desire discrepancy-associated distress and the relative importance and meanings of sexual desire were not assessed in the present study. Future studies should consider including these measures. Finally, in the present study, sexual desire discrepancy was investigated from an individual rather than a dyadic perspective, neglecting the dyadic interaction within which sexual desire discrepancy develops [5]. Therefore, although the individual perspective may provide an important key to understanding the discrepancy in sexual desire, future studies should consider the dyad as the unit of analysis rather than only one member of the couple and investigate not only the degree of discrepancy, but also the direction of the discrepancy.

Despite these limitations, the present study attempted to address the need proposed in the scientific literature to further investigate the predictors, correlates, and mediators underlying sexual desire discrepancy [5], by identifying the quality of dyadic sexual communication and sexual satisfaction as two important factors to be considered in the development of clinical and research guidelines to support individuals complaining of sexual desire discrepancy. The European Society for Sexual Medicine (ESSM) [5] has reported improving sexual communication between partners as one of the treatment options for sexual desire discrepancy; however, this treatment suggestion is mainly based on clinical rather than research experiences [5]. Thus, the results of our study represent initial scientific evidence within the limits of sexual desire discrepancy research supporting the importance of adopting nonsexual strategies to rebalance desire discrepancy. Therefore, it is important for sexual health professionals to work on activating both partners erotically, expand the scenario of daily activities by breaking the daily routine, and carry out psychoeducation by providing a broader definition of sex and sexuality and normalizing the fluctuating nature of desire. This mediation model can be a key to reading and understanding for practitioners and researchers working in the field of sexual and relational health, enabling them to navigate the complexity of the dynamics and factors underlying perceived sexual desire discrepancy. In light of this model and the findings that have emerged, working first on the quality of sexual communication through the encouragement of more open sexual communication in which partners share their sexual desires, fantasies, and concerns, is important for improving the quality of sexual satisfaction and, consequently, perceived sexual desire discrepancy [5]. This model can also be important in clarifying to partners the misinterpretations, beliefs, and biased perceptions that can often arise regarding the other partner’s desire and cause unnecessary discomfort. In addition, sexual health specialists should stimulate discussion to explore how each partner perceives and defines sex and whether they share the same view of it [70,71]. Clarifying this could be critical, as individuals are often focused on a narrow conceptualization of sex and the sexual relationship itself while losing sight of other sexual and nonsexual elements that are important to desire [71]. Therefore, interventions directed at balancing sexual desire discrepancy should aim to encourage quality dyadic sexual communication based on a mutually satisfying agreement on sexual and relational acts and interactions, rather than increasing sexual frequency [70,71,72].

## 5. Conclusions

In conclusion, the present study makes an important contribution to the scarce (albeit growing) and complex literature on the topic. To the best of our knowledge, as the first study to examine the effect of dyadic sexual communication and sexual satisfaction on the individual degree of perceived sexual desire discrepancy, the results provide initial evidence that the quality of dyadic sexual communication and sexual satisfaction appear to play a key role in perceived incongruence between one’s own sexual desire and that of one’s partner. Researchers and clinicians should continue to explore the relational dynamics underlying sexual desire discrepancy, while not losing sight of the subjective experience and complex nature of sexual desire.

## Figures and Tables

**Table 1:** Summary for intercorrelations, means, and standard deviation for scores on dyadic sexual communication, sexual satisfaction, and degree of perceived sexual desire discrepancy.

Variables	M	SD	1	2	3	4	5	6	7	8	9	10
1. Quality of dyadic Sexual communication	63.14	13.07	1									
2. Sexual satisfaction	21.73	6.06	0.61 **	1								
3. Degree of perceived sexual desire discrepancy	1.50	1.54	−0.35 **	−0.38 **	1							
4. Gender	-	-	−0.06	−0.00	0.03	1						
5. Age	28.96	8.63	−0.23 **	−0.12 *	0.14 **	0.21 **	1					
6. Sexual orientation	-	-	0.012	0.04	0.01	0.18 **	0.05	1				
7. Relationship duration	6.03	6.29	−0.27 **	−0.11 *	0.19 **	0.03	0.62 **	−0.05	1			
8. Having a child/children	-	-	−0.19 **	−0.12 *	0.10	−0.05	0.50 **	−0.09	0.53 **	1		
9. Desire to have a child/children	-	-	0.15 **	0.09	−0.15 **	−0.02	−0.46 **	−0.11 *	−0.35 **	−0.31 **	1	
10. Social desirability	7.83	2.35	0.16 **	0.21 **	−0.14 **	0.03	0.10	−0.07	0.01	0.06	−0.01	1

Note. Gender: 1 = female and 2 = male; sexual orientation: 1 = heterosexual and 2 = non-heterosexual; having a child/children: 0 = no and 1 = yes; desire to have a child/children: 0 = no and 1 = yes. * *p* < 0.05; ** *p* < 0.01.

**Table 2:** Results of the three mediation models.

	Degree of Perceived Sexual Desire Discrepancy						Sexual Satisfaction		
	Model 1			Model 3			Model 2		
	*β*	SE	95% CI	*β*	SE	95% CI	*β*	SE	95% CI
Constant	0.00	0.05	[−0.10, 0.10]	0.00	0.05	[−0.09, 0.09]	0.00	0.04	[−0.08, 0.08]
Quality of dyadic sexual communication	−0.31 ***	0.05	[−0.41, −0.20]	−0.14 *	0.06	[−0.26, −0.02]	0.61 ***	0.04	[0.52, 0.69]
Sexual satisfaction	-	-	-	−0.27 ***	0.06	[−0.39, −0.15]	-	-	-
Gender	0.02	0.05	[−0.09, 0.12]	0.02	0.05	[−0.08, 0.12]	0.03	0.04	[−0.06, 0.11]
Age	−0.02	0.07	[−0.16, 0.12]	−0.03	0.07	[−0.17, 0.10]	−0.05	0.06	[−0.16, 0.07]
Sexual orientation	0.00	0.05	[−0.10, 0.10]	0.01	0.05	[−0.08, 0.11]	0.05	0.04	[−0.04, 0.13]
Relationship duration	0.10	0.07	[−0.03, 0.23]	0.13*	0.07	[0.01, 0.26]	0.11 *	0.06	[0.00, 0.22]
Having a child/children	−0.02	0.06	[−0.14, 0.10]	−0.03	0.06	[−0.15, 0.08]	−0.05	0.05	[−0.15, 0.05]
Desire to have a child/children	−0.09	0.06	[−0.20, 0.02]	−0.08	0.05	[−0.19, 0.02]	0.01	0.05	[−0.08, 0.10]
Social desirability	−0.09	0.05	[−0.19, 0.01]	−0.06	0.05	[−0.15, 0.04]	0.12 **	0.04	[0.04, 0.21]
$R^{2}$	0.15 ***			0.19 ***			0.40 ***		
F	7.84			9.56			29.81		

*Note.* Gender: 1 = female and 2 = male; sexual orientation: 1 = heterosexual and 2 = non-heterosexual; having a child/children: 0 = no and 1 = yes; desire to have a child/children: 0 = no and 1 = yes. * *p* < 0.05. ** *p* < 0.01. *** *p* < 0.001.

## Data Availability

The data presented in this study are available on request from the corresponding author. The data are not publicly available due to privacy reasons.
